# Treatment of Cognitive Deficits and Behavioral Symptoms Following COVID-19-Associated Autoimmune Encephalitis With Intravenous Immunoglobulin: A Case Report and Review of the Literature

**DOI:** 10.7759/cureus.51071

**Published:** 2023-12-25

**Authors:** Suniya Naeem, Sarah M Oros, Christian S Adams, Gopalkumar Rakesh

**Affiliations:** 1 Child Psychiatry, Washington University School of Medicine, St. Louis Children's Hospital, St Louis, USA; 2 Psychiatry/Internal Medicine, University of Kentucky College of Medicine, Lexington, USA; 3 Psychiatry, University of Kentucky College of Medicine, Lexington, USA

**Keywords:** behavioral disorder, intravenous immunoglobulin therapy, encephalitis, cognitive deficits, covid-19

## Abstract

Coronavirus disease 2019 (COVID-19) is associated with long-term neuropsychiatric sequelae. We describe a 60-year-old male patient's history and symptom trajectory encompassing the development of behavioral symptoms and cognitive deficits following pneumonia and subsequent autoimmune encephalitis associated with COVID-19. We also describe changes in these facets with correlative changes in his immunological parameters after both acute intravenous immunoglobulin (IVIG) therapy and chronic periodic IVIG therapy every two weeks over the course of two years. ​​​​​​We review the literature on the treatment of long COVID-19 symptoms spanning cognitive and behavioral domains. In addition, we also elucidate current literature on the role of IVIG infusions for these symptoms using our patient's presentation and improvement in symptoms as an illustrative example.

## Introduction

Coronavirus disease 2019 (COVID-19) is a disease that primarily targets the respiratory system. Cross-sectional and longitudinal studies have identified additional acute and chronic effects on other organ systems [1]. While neurological syndromes like encephalitis and encephalopathy are acute complications of COVID-19 infection [2], long-term sequelae of these complications include behavioral symptoms and cognitive impairment [3,4]. This makes it pertinent to diagnose and treat these acute complications, while also optimizing treatments for long-term sequelae [3]. Case reports have demonstrated the efficacy of intravenous immunoglobulin (IVIG) therapy in treating neurological and behavioral symptoms associated with COVID-19 [5]. To date, there are no longitudinal follow-up studies examining treatment outcomes for patients experiencing neurological and behavioral sequelae of COVID-19-associated autoimmune encephalitis.

Here, we present the symptoms and long-term trajectory of a 60-year-old high-functioning male patient who presented with altered mental status, cognitive deficits, and behavioral disturbances following COVID-19. He was diagnosed with seronegative autoimmune encephalitis related to COVID-19 based on his clinical symptoms and neuroimaging. A brief summary of the patient's acute presentation and improvement in symptoms with a five-day course of IVIG has been published previously [6]. A summary of his immunological parameters related to COVID-19 and improvement in these parameters with periodic IVIG therapy has also been detailed elsewhere [7]. This case series elucidates how periodic IVIG therapy has been effective in the treatment of cardiac and pulmonary symptoms in addition to abnormal immunological parameters associated with long COVID-19 [7]. 

We present a comprehensive description and trajectory of the patient's behavioral symptoms following COVID-19, supported by neuroimaging and neuropsychological evaluations. We also describe changes in these facets with correlative changes in his immunological parameters after both acute IVIG therapy and chronic periodic IVIG therapy over the course of two years. ​​​​​​We review the literature on treatment of long COVID-19 symptoms spanning cognitive and behavioral domains. In addition, we also elucidate current literature on the role of IVIG infusions for these symptoms using our patient's presentation and improvement in symptoms as an illustrative example. 

## Case presentation

Our patient is a 60-year-old male who presented to the hospital in April 2020 with a two-day history of cough, sore throat, and dyspnea following COVID-19 exposure. The nasopharyngeal swab was positive for COVID-19 RNA using a polymerase chain reaction (PCR) test. His chest X-ray was significant for faint bibasilar airspace opacities and right mid lung peripheral faint opacities. He had tachycardia, tachypnea, and fevers (up to 100.8 F) and required up to three liters of oxygen via a nasal cannula to maintain 95% saturation. He finished a five-day course of hydroxychloroquine and azithromycin with improvement in symptoms and was deemed stable for discharge on day 14 after inpatient hospital admission. He was not given any steroids during this admission. 

He then returned to the emergency room (ER) less than two months later with a four-day history of worsening mental status. He was noticed by his colleagues at work to have significant behavioral changes including emotional lability, paranoia about his safety, and spontaneous crying spells. He found it difficult to form coherent sentences or follow conversations. He also developed difficulty writing with changes in his handwriting. He would also get combative with his wife without reason and found it difficult to perform activities of daily living (ADLs), which precipitated the repeat visit to the ER.

On presentation to the ER, he exhibited auditory and visual hallucinations (comprising sounds like "bangs or cracks" and visual perceptual deficits of images seeming "holographic or lumpy"), in addition to disinhibited behavior toward his spouse. His symptom burden also included a lack of orientation to time, place, or person. Serum thyroid stimulating hormone (TSH), C-reactive protein (CRP), lactate dehydrogenase (LDH), cyclic citrullinated peptide (CCP) IgG, antistreptolysin (ASO) titer, and heavy metal screen were unremarkable. Serum anti-nuclear antibody was positive (1:160, speckled pattern). Lumbar puncture showed newly elevated cerebrospinal fluid (CSF) protein (54 mg/dl with normal range being 15-45 mg/dl), negative CSF pleocytosis, negative CSF SARS-CoV-2, negative CSF Lyme antigen, anti-NMDA antibody titer < 1:1, and unremarkable CSF multiple sclerosis panel. Computed tomography (CT) brain was unremarkable except for a chronic infarct in the right medial thalamus. EEG also showed diffuse slowing suggestive of encephalopathy. Serum autoimmune panel labs were negative. His Montreal Cognitive Assessment (MoCA) score was 17/30 with points lost on delayed recall (0/5), visuospatial ability (1/3), trail making (0/1), serial subtraction (1/3), orientation (4/6), and digit span backward (0/1).

**Figure 1:**
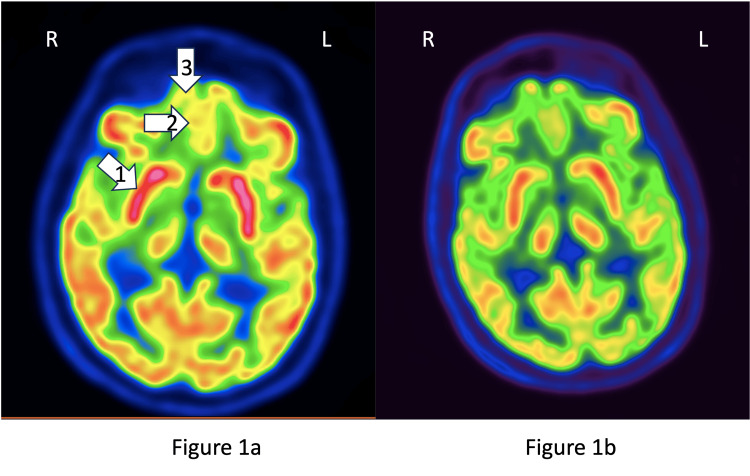
PET FDG Scans In a PET scan, colors showing decreasing order of metabolic activity are red, yellow, green, and blue. 1a shows areas of hypermetabolism, indicated by red/red-white shade for the basal ganglia (1), yellow shade for the olfactory cortex (2), and superior/middle frontal gyri (3) leading to the diagnosis of autoimmune encephalitis. Following the five-day course of IVIG, these areas (1b) showed hypometabolism (trending towards normal) as indicated by a decrease in the intensity of red shade for basal ganglia (1), green shade for olfactory cortex (2), and green-yellow shade for superior and middle frontal gyri (3). PET FDG: Positron emission tomography fluorodeoxyglucose

While consensus guidelines recommend MRI brain (with and without contrast) with EEG for workup of autoimmune encephalitis, a fluorodeoxyglucose positron emission tomography (FDG-PET) scan is recommended if MRI is not conclusive and clinical suspicion remains high [8]. Upon recommendation from psychiatry and neurology consult teams, he underwent a PET scan of the brain which showed hypermetabolism in bilateral basal ganglia, superior and middle frontal gyro, and olfactory cortex (Figure 1a) consistent with the early pattern of autoimmune encephalitis [9]. Based on consensus recommendations from the patient's primary internal medicine team, and neurology and psychiatry consult teams, he was started on a five-day course of intravenous immunoglobulin (IVIG) therapy. He was also started on quetiapine and dose titrated to 300 mg for behavioral symptoms encompassing frequent instances of paranoid ideation, and insomnia. He was also initiated on sertraline 25 mg for irritability and emotional regulation. There was considerable improvement in his mental status after the first day of IVIG therapy with incremental improvement in his orientation, behavior, and ability to perform ADLs after every day of IVIG therapy. A follow-up FDG-PET scan one week later showed an interval global decrease in cortical brain metabolism, more pronounced in bilateral basal ganglia, superior and middle frontal gyro and olfactory cortices, reflecting a change towards baseline and reflecting treatment response to IVIG (Figure 1b). He was stabilized on 100 mg of quetiapine to treat his visual and auditory hallucinations and insomnia. The 'paranoia' seemed to encompass being anxious about his safety from people he knew. Although he wasn't able to verbalize what the threat to his life was, this anxiety resulted in him becoming irritable with his wife and treatment teams. The sertraline was titrated to 100 mg to treat irritability and for emotional regulation. At the time of discharge on day 14, his MoCA score improved to 26/30, with points lost of delayed recall (3/5) and serial subtraction (2/3) and orientation (5/6).

He was brought back to the hospital four months later due to a recurrence of altered mental status, disinhibited behavior, and confusion. His mental status and symptoms were deemed to be considerably similar to those during previous admissions and based on consensus recommendations from neurology and psychiatry teams, he was given another five-day course of IVIG with dramatic improvement in symptoms. Repeat MoCA showed a score of 26/30 with 2 points lost on delayed recall, 1 point lost on verbal fluency, and 1 on orientation (date). His sertraline was titrated to 200 mg at the time of discharge.

**Figure 2:**
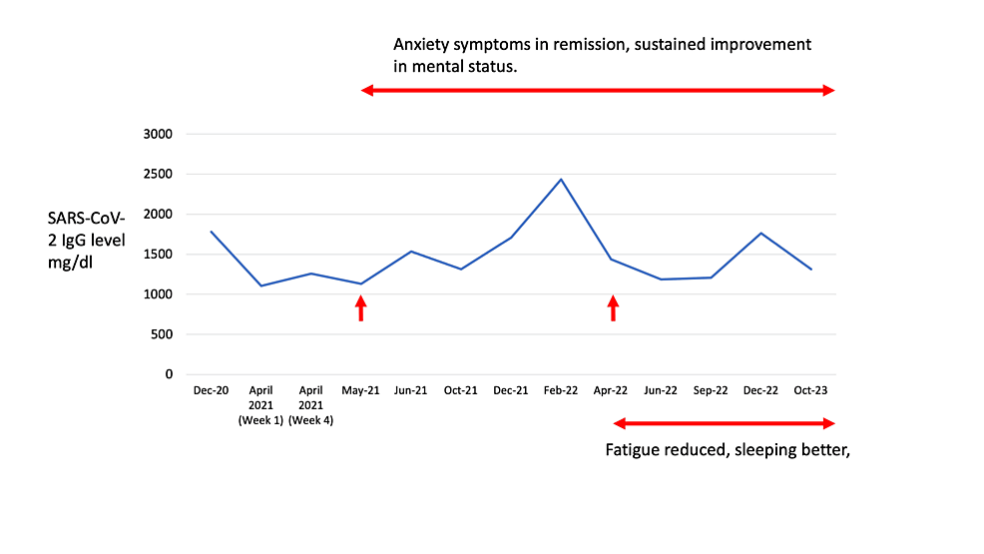
SARS-CoV-2-IgG Level Over Time We observed a sustained reduction in the patient's IgG level after starting IVIG infusions every two weeks, correlated with remarkable improvement in mental status and anxiety symptoms. Despite the presence of cognitive deficits, the patient demonstrated sustained improvement in alertness and orientation to time, person, and place. Over the course of time, he started sleeping better with sustained improvements in emotional regulation, fatigue, and irritability. Albeit a spike in February 2022 associated with COVID-19 vaccination, he continued to demonstrate improvement in the facets detailed above. IVIG: Intravenous immunoglobulin

After discharge, he was referred to the Immunology clinic, given that his SARS-CoV-2 IgG was elevated (1781 mg/dl) before IVIG therapy during this admission (Table 1). His immunology team recommended providing the patient with 0.5mg/kg IVIG every two weeks [7]. The goal of these infusions was to address elevated SARS-CoV-2 IgG which correlated with worsening in his mental status and fatigue. These facets would resolve following IVIG therapy but worsen by the end of two weeks. This IVIG infusion regimen was tested out for three months initially which resulted in sustained improvement in his mental status (decreased confusion and being oriented to time, place, and person). At the end of three months, the team decided to continue the IVIG infusions after observing sustained improvement in his behavior and mental status [7]. Although initial IVIG infusions decreased confusion acutely at the end of every three weeks, improvements in anxiety symptoms and emotional regulation were also noticed toward the end of the three-month period which advocated for continuing the infusions. Table 1 shows his monthly serum immunoglobulin values (IgG, IgA, and IgM) estimated before the infusions were administered, with correlative behavioral symptoms. As shown in Table 1 and Figure 2, his IgG would elevate by the end of the two-week period. He continued IVIG therapy every two weeks all through 2021-2023 till October 2023 when he was transitioned to once every four weeks. He continues to receive IVIG every four weeks now.

**Table 1:** Immunological Profile With Behavioral Symptoms ADLs: Activities of daily living

Date	IgG	IgA	IgM	Behavioral symptoms and cognitive deficits
December 2020	1781	86	46	Anxious about personal safety, instances wherein he is ‘frozen’ because of anxiety. Confused whether its day or night at times.
April 2021 (Week 1)	1106	64	28	Anxiety continuing to be present, frustrated when unable to perform ADLs or tasks requiring more mental effort.
April 2021 (Week 4)	1260	72	31	Able to reflect when frustrated, reduction in anxiety. Alert and oriented.
May 2021	1130	68	30	Cognitive deficits persist, anxiety symptoms in remission
June 2021	1536	74	28	Increased fatigue, anxiety stable, sleeping better.
October 2021	1314	75	36	Traveled abroad, behavioral symptoms stable
December 2021	1709	80	32	Fatigue continues to be present, otherwise stable
February 2022	2434	84	41	Received COVID vaccine doses in December 2021 and January 2022.
April 2022	1435	75	35	Cognitive deficits persist and have plateaued, has not returned to previous employment. Anxiety symptoms in remission, sleeping through the night. Fatigue has improved.
June 2022	1188	75	35	
September 2022	1207	77	35	
December 2022	1762	79	51	
October 2023	1312	79	60	

During the course of these periodic IVIG infusions in 2021-2023, his anxiety, instances of irritability, and insomnia improved significantly despite no changes in the doses of his psychotropic medications (Table 1). He was on sertraline 200 mg and quetiapine 100 mg all along. During a recent appointment in October 2023, his wife reported that the anxiety had been stable for two years, and he sleeps ~ 8 hours every night. His emotional regulation has been optimal albeit instances of frustration secondary to cognitive deficits. We performed neuropsychological evaluation to quantify his cognitive deficits in 2022 and 2023 (Table 2). Both evaluations were performed within 4-5 days of IVIG infusions. He was noted to have severe impairment in verbal memory and cognitive interference (measured with a Stroop task) during both evaluations (Table 2). During both evaluations, he was also noted to have suboptimal performance on working memory and processing speed tasks (Table 2), correlating with his difficulties managing his financial affairs and performing complex tasks, while being able to perform ADLs. Although both neuropsychological evaluations showed deficits in these domains (Table 2), remarkable improvements in his mental status, anxiety, irritability, insomnia, and emotional regulation advocated for continuing the IVIG infusions. 

**Table 2:** Neuropsychological Evaluations *WAIS-IV: Wechsler Adult Intelligence Scale-Fourth Edition; HVLT: Hopkins Verbal Learning Test; WMS-IV: Wechsler Memory Scale-Fourth Edition; BVMT-R: Brief Visuospatial Memory Test-Revised; RCFT: Rey Complex Figure Test and Recognition Trial; SDMT: Symbol Digit Modalities Test; BNT-2: Boston Naming Test-Second Edition; MAE: Multilingual Aphasia Examination; JLO: Benton Judgement of Line Orientation, form V; BDI-II: Beck Depression Scale-Second Edition; BAI: Beck Anxiety Inventory.

2022				2023			
Learning and memory							
	Domain	Raw Score	Percentile	Interpretation	Raw Score	Percentile	Interpretation
WAIS-IV Matrix Reasoning	Non-verbal reasoning, perceptual organization, and visual intelligence	22	95	Superior	16	63	Average
HVLT 1-3 Total	Hopkins Verbal Learning Test (HVLT) to assess immediate and delayed verbal recall. The test also calculates a verbal retention score and verbal recognition discrimination index.	19	2	Severely Impaired	21	10	Below Average
HVLT LD Free		6	2	Severely Impaired	8	21	Below Average
HVLT Retention		86%	34	Average	114%	96	Superior
HVLT Recognition		12	84	Above Average	7	<1	Severely Impaired
WMS-IV LM I	Logical memory (LM) subtest of the Wechsler Memory Scale (WMS) to measure verbal episodic memory (immediate recall, delayed recall, and delayed recognition)	30	75	Average	21	25	Average
WMS-IV LM II		25	75	Average	16	25	Average
WMS-IV LM Recognition		28	>75	Above Average	25	50-75	Average
BVMT-R 1-3 Total	Brief Visuospatial Memory Test-Revised (BVMT-R) to measure visuospatial learning and memory.	22	46	Average	20	34	Average
BVMT-R LD Free		9	62	Average	7	24	Below Average
BVMT-R Retention		90%	>16	WNL	88%	>16	WNL
BVMT-R Recognition		40	6-10	Borderline Impaired	3	3-5	Impaired
RCFT Immediate Recall	Rey Complex Figure Test (RCFT) to measure visuospatial recall memory, recognition memory, and constructional ability.	20.5	79	Above Average	13.5	27	Average
RCFT Recognition		19	24	Below Average	16	1	Severely Impaired
Attention, Processing Speed, & Executive Functioning							
WAIS-IV DS Total	Digit Span (DS) subtest from the Wechsler Adult Intelligence Scale-Fourth Edition. Comprises Digit Span Forward (DSF), Digit Span Backward (DSB) and Digit Span Sequencing (DSS) to measure working memory.	19	9	Below Average	18	9	Below Average
WAIS-IV DSF		8	25	Average	8	25	Average
WAIS-IV DSB		5	9	Below Average	6	16	Below Average
WAIS-IV DSS		6	16	Below Average	4	5	Impaired
SDMT Written	Symbol Digit Modalities Test (SDMT) to measure processing speed.	45	38	Average	37	10	Below Average
SDMT Oral		45	14	Below Average	Not done	Not done	Not done
Trails A	Fluid intelligence and processing speed.	43	12	Below Average	36	24	Below Average
Trails B		75	27	Average	91	14	Below Average
Stroop Word	The test measures the ability to inhibit cognitive inteference	55	<1	Severely Impaired	50	<1	Severely Impaired
Stroop Color		48	<1	Severely Impaired	39	<1	Severely Impaired
Stroop C-W		29	6	Borderline Impaired	22	2	Severely Impaired
Language, Visuospatial, & Motor Skill							
Letter Fluency		36	21	Below Average	26	6	Impaired
Semantic Fluency		16	12	Below Average	14	5	Impaired
BNT-2	Naming and word retrieval	57	38	Average	58	66	Average
MAE Sentence Rep	The sentence repetition subtest of the multilingual aphasia examination (MAE) measures oral expression.	12	43	Average	10	15	Below Average
JLO (adjusted)	Benton Judgement of Line Orientation (JLO) measures visuospatial judgment.	14	76	Above Average	15	89	Above average
Benton Visual Form Discrimination	Visual discrimination and visual recognition memory	32	85	Above Average	27	13	Below Average
RCFT-Copy	Visuospatial construction	33	>16	WNL	31	11-16	Below Average

## Discussion

Our patient fulfilled the diagnostic criteria for autoimmune encephalitis in having altered mental status encompassing behavioral changes and cognitive deficits, PET scan changes and exclusion of alternative causes [9]. Our patient did not receive steroids when hospitalized initially with pneumonia related to COVID-19 infection. While the RECOVERY trial did find a positive effect of steroids on mortality for patients on mechanical ventilation for COVID-related respiratory distress [10], this effect was not observed when patients were monitored for 90 days [11,12]. Consequently, the short-term and long-term benefits of steroids in COVID-19 are an area of ongoing research [13]. Hence, it is plausible that the lack of treatment with steroids may have contributed to the development of autoimmune encephalitis and consequent cognitive deficits [14]. The association between long-term cognitive deficits and pulmonary function continues to be an area of active research [14].

Conceptualizing the trajectory of symptom development, we could postulate that the cognitive deficits developed initially with the altered mental status that led to the diagnosis of autoimmune encephalitis. Unfortunately, we do not have neuropsychological evaluations from 2020 or 2021 to support this assumption. The IVIG infusions delivered over five-day periods during his two initial admissions resulted in dramatic improvement and resolution of the altered mental status. Progressive decline in his mental status following discharge from the latter admission led to an examination of his immunological profile.

The rationale for proposing biweekly infusions of IVIG was based on body weight dosing [7]. Although IVIG has been shown to partially or completely resolve a full spectrum of neurological syndromes following COVID-19 (Guillain Barre Syndrome, myositis, Miller Fisher Syndrome, polyradiculopathy, encephalitis, encephalopathy) [5], we did not come across any studies that utilized IVIG for treatment of behavioral or cognitive symptoms. There is one retracted case study wherein IVIG improved psychosis associated with autoimmune encephalitis [15]. 

Long-term IVIG therapy administered every 2-6 weeks over the course of years has demonstrated sustained remission of symptoms in other autoimmune spectrum disorders like chronic inflammatory demyelinating polyneuropathy, multifocal motor neuropathy (MMN), and myasthenia gravis [16]. We lack a consensus approach in deciding the frequency of dosing in these disorders. Nonetheless, there has been some effort in optimizing it using an algorithmic approach [17]. Response to IVIG varies mainly due to differences in catabolism and speed of diffusion into extracellular spaces [17]. 

More importantly, we also observed a steady improvement in his anxiety symptoms, insomnia, and emotional regulation with the periodic infusions. We could speculate that the quetiapine and sertraline contributed to this; however, the doses remained unchanged. Although no symptom scale ratings were obtained to measure anxiety symptoms, insomnia, and mood, the patient's wife who is his primary caregiver attested to significant improvement in these facets with IVIG infusions. Given the improvement in his behavioral symptoms and sleep with IVIG infusions, it is possible that these symptoms share neuroinflammatory pathophysiology with the cognitive symptoms, in the context of COVID-19 [18]. Neuroinflammation in the aftermath of COVID-19 is associated with neurological, and behavioral symptoms in addition to cognitive deficits [3,19]. 

Our patient's presentation fulfills criteria for long COVID-19 [3] with cognitive deficits. Common behavioral symptoms associated with long COVID-19 (in decreasing order of prevalence) include sleep disturbances, cognitive impairment, fatigue, anxiety symptoms, post-traumatic stress disorder (PTSD), and major depressive disorder [3]. Our patient had sleep disturbances, cognitive impairment, fatigue, and anxiety symptoms all of which except the cognitive impairment improved with the IVIG infusions. In addition, the IVIG infusions resulted in a sustained reduction in IgG levels, except for a spike associated with COVID-19 vaccination. In this context, it is pertinent to highlight the propensity of COVID-19 vaccination to trigger autoimmune CNS manifestations encompassing autoimmune encephalitis, pituitary apoplexy, and hypophysitis [20-22]. 

 Although it is possible that his other cognitive deficits improved over time with variable contributions from the IVIG infusions, verbal memory and cognitive interference (measuring with Stroop) were severely impaired during both evaluations. He had persisting working memory deficits as well. This is consistent with existing studies showing deficits in interference, verbal memory, and working memory to persist after the COVID-19 infection [10,23]. Treatment of behavioral symptoms and cognitive deficits associated with long COVID-19 continues to be an area of active research [3]. Given the improvement we witnessed in behavioral symptoms in our patient, we advocate for more studies using IVIG (open-label trials and randomized controlled trials with varying IVIG doses) to treat behavioral symptoms and cognitive impairment in long COVID-19. 

## Conclusions

In summary, this case report highlights the behavioral and cognitive long-term sequelae of COVID-19 infection, following the occurrence of COVID-19 pneumonia and COVID-19-associated autoimmune encephalitis. The behavioral sequelae improved significantly with IVIG therapy. The rationale and frequency of IVIG infusions were decided upon based on similar treatment strategies in other autoimmune disorders. We are unable to comment on improvement in cognitive symptoms with IVIG, due to the lack of neuropsychological testing data from 2020 and 2021. Nonetheless, deficits in verbal memory and cognitive interference persist despite periodic infusions. We hope for the manuscript to extend the literature on treating behavioral symptoms and cognitive deficits associated with long COVID-19. We advocate for more studies using IVIG (open-label trials and randomized controlled trials with varying IVIG doses) to treat behavioral symptoms and cognitive impairment in long COVID-19. 
